# Association between histogram parameters of T2 weighted MRI data, WHO grade, tumor proliferation index and selected molecular markers in low-grade glioma

**DOI:** 10.3389/fradi.2026.1730316

**Published:** 2026-01-29

**Authors:** Georg Gihr, Hans Henkes, Ali Khanafer, Mehdi Allouche, Oliver Ganslandt, Sebastian Johannes Müller, Walter Alexander Wohlgemuth, Stefan Schob

**Affiliations:** 1 Clinic for Neuroradiology, Katharinenhospital Stuttgart, Stuttgart, Germany; 2 Clinic for Neurosurgery, Katharinenhospital Stuttgart, Stuttgart, Germany; 3 Clinic for Neuroradiology, University Hospital Magdeburg, Magdeburg, Germany; 4 Department for Radiology, University Hospital Halle (Saale), Halle (Saale), Germany

**Keywords:** histogram analysis, histopathology, imaging biomarker, low-grade glioma, T2 weighted imaging, tumor heterogeneity

## Abstract

**Introduction:**

Conventional T2 weighted MRI sequences are part of the standard diagnostics in case of LGG and implemented in every MRI protocol for the first anatomical evaluation. Despite the excellent tissue contrast and spatial information, conventional radiological assessment of these images lacks the capacity of providing reliable information about underlying histopathology. Therefore, this retrospective investigation aimed to assess whole-tumor histogram analysis (HA) of T2 weighted MRI sequences for its ability to distinguish between WHO grade 1 and 2 gliomas, to predict the isocitrate dehydrogenase 1 (IDH 1) gene mutation and the methylguanine-DNA methyl-transferase (MGMT) promoter methylation status, to differentiate oligodendrogliomas from diffuse astrocytomas, and to predict the proliferative potential using the Ki-67 proliferation index.

**Methods:**

Signal intensities of T2 weighted pre-surgical MRI data of 53 LGG patients were used for histogram-profiling. WHO-grade, Ki-67 expression, IDH 1 mutation and MGMT promoter methylation status were evaluated. Comparative and correlative statistics were used to investigate possible associations between HA parameters and neuropathology.

**Results:**

Statistically significant distinctions between WHO grade 1 and grade 2 gliomas were observed for T2 SI Entropy (*p* = 0.001). Furthermore, T2 SI Entropy was significantly higher in IDH 1-mutated gliomas (*p* = 0.015) and correlated significantly with the Ki-67 proliferation index (*r* = 0.341, *p* = 0.019). Noteworthy distinctions between gliomas with MGMT promoter methylation and those without were discerned for SImin (*p* = 0.019). No significance could be detected comparing SI histogram parameters between oligodendrogliomas and diffuse astrocytomas.

**Conclusion:**

Our investigation demonstrates the potential of T2 SI Entropy in distinguishing grade 1 from grade 2 gliomas and in reflecting the proliferative activity denoted by Ki-67 expression, therefore being a promising HA feature for assessing tumor heterogeneity.

## 1 Introduction

Gliomas represent a heterogeneous group of primary central nervous system (CNS) tumors arising from glial cells, encompassing astrocytic, oligodendroglial, and ependymal subtypes, with diverse histopathological and molecular characteristics. In contrast to non-CNS neoplasms the classification and prognosis of gliomas are regularly determined by World Health Organization (WHO) grading, integrating histopathologic characteristics, biological tumor behavior and increasingly molecular biomarkers (1). Low-grade gliomas (LGG), categorized as WHO grade 1 and 2, typically manifest relatively slow growth and a less aggressive clinical course compared to high-grade gliomas (HGG, WHO grade 3 and 4). Subtypes within LGG include pilocytic astrocytoma, diffuse astrocytoma, and oligodendroglioma, collectively constituting approximately 15%–16% of all primary brain tumors (2).

Conventional anatomical magnetic resonance imaging (MRI) sequences, namely T2, fluid-attenuated inversion recovery (FLAIR) and T1 weighted images (pre- and post-contrast) are the first-line technique in terms of identifying the location of the tumor and its expansion into adjacent brain areas (3). Nevertheless, conventional MRI images are often morphologically ambivalent among the different LGG subtypes, even when using gadolinium-based contrast agents, and their routinely applied qualitative radiological assessment does not reliably allow to draw conclusions about the underlying histopathology. To overcome this limitation advanced MRI techniques such as diffusion weighted imaging (DWI), perfusion weighted imaging (PWI) and MR spectroscopy (MRS) have been long used in addition to routine anatomical evaluation (4) and especially assessed in a quantitative approach they helped to improve the diagnostic accuracy (5).

A frequently used method for such a quantitative evaluation of MRI data is histogram analysis (HA), a statistical technique to examine the distribution of data values within a dataset. By analyzing the shape and other characteristics of the MRI data histogram, represented by different histogram parameters, it is possible to gain valuable insights into the underlying tissue properties, thereby enhancing the MRI assessment of tumor heterogeneity (6). Numerous histogram-based investigations, especially of advanced MRI data such as DWI and PWI, have been presented in recent years with promising results in terms of predicting tumor grade, estimating the proliferative activity of the lesion at hand and delivering further prognostic information (7–19). Notably, studies have demonstrated that HA, even when applied to conventional anatomical MRI sequences, is capable of offering indications of biological tumor characteristics and histopathology across different tumor entities (20–25). This observation underscores the potential for HA parameters to be MRI sequence independent biomarkers. But before routinely integrating them as a component within a contemporary quantitative approach, not only applying to advanced MRI data but also to widely available conventional sequences, further investigations to clarify which of these HA parameters are the most promising need to be conducted.

Therefore, the objective of this retrospective investigation was to assess the capacity of whole tumor HA of T2 weighted MRI sequences to i) distinguish between WHO grade 1 and WHO grade 2 gliomas, ii) anticipate the isocitrate dehydrogenase 1 (IDH 1) mutation and methylguanine-DNA methyl-transferase (MGMT) promoter methylation status, iii) differentiate oligodendrogliomas from diffuse astrocytomas, and iv) predict the proliferative potential of the neoplasms, as indicated by the Ki-67 proliferation index.

## 2 Materials and methods

### 2.1 Patients

Our institutional Radiological Information System (RIS) was systematically searched for patients diagnosed with low-grade glioma and primary brain tumors. A total of 61 patients, who underwent diagnostic biopsy or surgical removal of tumors with subsequent neuropathological assessment within our hospital between January 2012 and January 2017 were identified. Tumor classification and grading was conducted according to the 2016 WHO classification of CNS tumors. Inclusion criteria mandated patients to have undergone pretreatment Magnetic Resonance Imaging (MRI) scans with sufficient T2 weighted imaging data. Patients with an artificial image data set, mostly due to motion related artifacts, or patients with intratumoral hemorrhage were excluded.

Ultimately, 53 patients qualified for our retrospective analysis, comprising 11 pilocytic astrocytomas WHO 1, 31 diffuse astrocytomas WHO 2 and 11 oligodendrogliomas WHO 2.

The WHO 1 pilocytic astrocytoma group consisted of 5 females and 6 males, with a mean age of 24 years. The WHO 2 diffuse astrocytoma group comprised 15 females and 16 males, with a mean age of 41 years. In the WHO 2 oligodendroglioma group were 5 females and 6 males with a mean age of 43 years. Among the cohort, 33 patients exhibited an IDH 1 mutation, while 15 patients had an IDH 1 wildtype status (IDH 1 mutation status was unavailable for 5 patients). MGMT promoter methylation was observed in 23 patients, and 11 patients presented with an unmethylated MGMT promoter (MGMT promoter methylation status was unavailable for 19 patients). 4 patients lacked available Ki-67 proliferation index data and were consequently excluded from the correlation analysis.

### 2.2 MRI protocol

All patients received a brain MRI scan using a 1.5T device (MAGNETOM Aera or MAGNETOM Symphony Tx/Rx CP head coil, Siemens, Erlangen, Germany). 47 out of 53 patients were examined with the MAGNETOM Area system using the same protocol and set of parameters. The imaging protocol included the following sequence:

Axial T2 weighted (T2w) turbo spin echo (TSE) sequence (TR/TE: 5390/99, flip angle: 150 °, slice thickness: 5 mm, acquisition matrix: 512 × 291, field of view: 230 × 187 mm).

All digitalized MRI images were analyzed by two experienced radiologists (DHR, SS) on a PACS workstation (Impax EE R20 XII).

### 2.3 Histogram analysis of T2 SI volumes

T2 weighted turbo spin echo (TSE) images were extracted from our institutional archive in Digital Imaging and Communications in Medicine (DICOM) format through the aforementioned AGFA PACS. Subsequently, comprehensive lesion histogram profiling was conducted using a DICOM image analysis tool developed by N.G. using MATLAB (The Mathworks, Natick, MA, USA). In the MATLAB function histogram, the bin width was determined using the auto setting, which is based on the Freedman–Diaconis rule. No additional normalization or interpolation was performed. T2 weighted images were loaded into a graphical user interface (GUI) to annotate the neoplastic lesions in each patient across all respective MRI sections. The tumor segmentation process was manually performed in consensus by two experienced neuroradiologists. Cystic components and adjacent edema were excluded.

The entire lesion histogram profile of T2 signal intensities (SI) was then systematically calculated, yielding a comprehensive set of parameters, including SImean, SImin, SImax, SIp10, SIp25, SIp75, SIp90, SImodus, SImedian, SI standard deviation (SD), Skewness, Kurtosis, and Entropy.

### 2.4 Neuropathology

All tumor specimens underwent neuro-histological verification of the diagnosis. Tumor samples, acquired through stereotactic biopsy, partial, or complete resection, were subjected to formalin fixation and paraffin embedding for subsequent histopathologic diagnostics, immunohistochemistry, and polymerase chain reaction (PCR) sequencing. The embedded samples were sectioned at a thickness of 3 µm and stained with hematoxylin and eosin (H&E).

Immunohistochemistry involved the use of specific antibodies against IDH1-R132H (dilution 1:20, product no. DIA-H09; Dianova, Hamburg, Germany) and Ki67 (dilution 1: 800; M7240; Dako Denmark A/S, Glostrup, Denmark). Histopathological images were digitized using a Leica microscope equipped with a DFC290 HD digital camera and LAS V4.4 software (Leica Microsystems, Wetzlar, Germany). Sections designated for immunohistochemistry and PCR sequencing underwent histological analysis to ascertain the presence of viable tumor infiltration while ensuring the absence of necrotic areas and hemorrhage. A positive result for IDH 1 immunohistochemistry was determined by strong cytoplasmic staining. The tumor proliferation index was calculated by dividing the number of specifically stained (Ki-67 positive) cell nuclei by the total number of nuclei, with the area displaying the highest number of positive cell nuclei selected in each case.

Fluorescence *in situ* Hybridization (FISH) was used to detect 1p/19q codeletion using dual-color probes targeting 1p36/1q25 and 19q13/19p13.

For the assessment of the MGMT gene's methylation status, tumor DNA was isolated from micro-dissected 10 µm-thick sections from paraffin-embedded tissue blocks using the Maxwell® RSC FFPE Plus DNA Kit AS1720 (Promega, Madison, WI, USA) and the Maxwell® RSC Instrument (Promega). The isolated DNA underwent bisulfite treatment using the EpiTect® Bisulfite Kit (QIAGEN, Hilden, Germany) following the manufacturer's procedures. The bisulfite-converted DNA was then amplified in a PCR reaction, and the methylation status was determined by pyrosequencing using the Therascreen MGMT Pyro® Kit (QIAGEN), targeting 4 CpG islands (chromosome 10, Exon 1, range 131265519–131265537, CGACGCCCGCAGGTCCTCG). A methylation percentage of 10% or higher was considered positive for methylation.

### 2.5 Statistical analysis

Statistical analyses, including graphical representation, were conducted utilizing GraphPad Prism 9 (GraphPad Software, San Diego, CA, USA). In the initial phase, a comprehensive examination of T2 SI data and histopathological information was undertaken through descriptive statistics. Subsequently, Gaussian distribution of the data was assessed using the Shapiro–Wilk Test. A T-Test was executed to compare normally distributed parameters derived from T2 SI histogram profiling between WHO 2 and WHO 2 gliomas. Additionally, unpaired T-Tests were employed to compare normally distributed T2 SI histogram profiling parameters between IDH 1-mutated and IDH 1-wildtype gliomas, between MGMT promoter-methylated and unmethylated gliomas as well as between oligodendrogliomas and diffuse astrocytomas. For parameters exhibiting a non-Gaussian distribution, Mann–Whitney-U Tests were conducted.

Correlation analyses for normally distributed parameters utilized the Pearson Correlation Coefficient, while Spearman-Rho Rank-Order Correlation was employed in cases of non-Gaussian distribution.

A significance threshold of *p*-values < 0.05 was universally adopted.

Finally, the accuracy of T2 SI histogram profiling was assessed through receiver operating characteristics (ROC) curve analysis. The corresponding area under the curve (AUC) was calculated, and Youden's Index was determined for parameters with optimal test accuracy to estimate potential cut-off values.

## 3 Results

**Figure 1:**
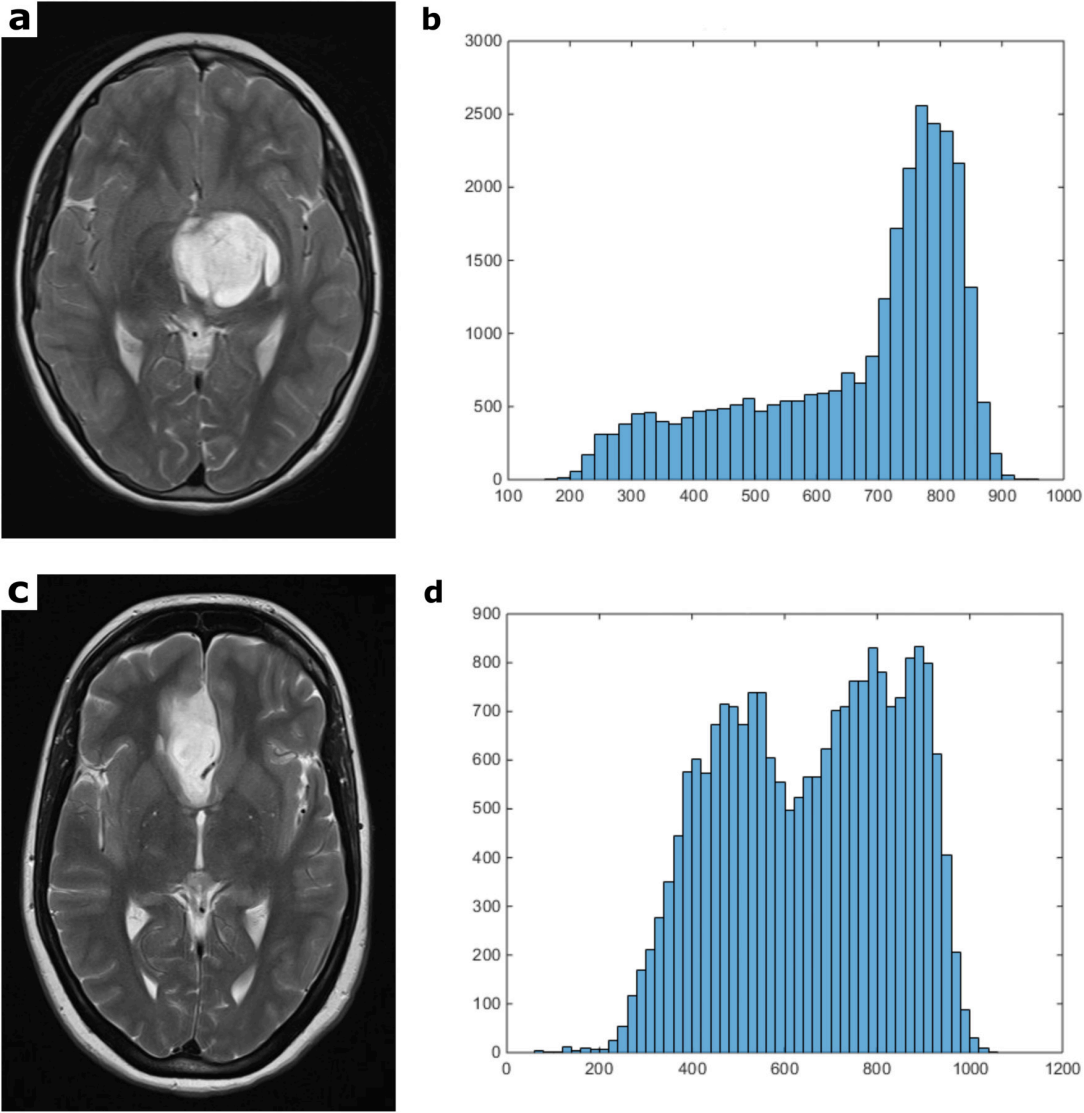
Figure 1 compares representative T2 weighted MRI sections and the corresponding whole tumor SI histogram of a grade 1 **(a,b)** and a grade 2 glioma **(c,d)**. The first image of the upper case shows a T2 weighted turbo-spin-echo (TSE) sequence of a pilocytic astrocytoma (WHO grade 1), located in the left thalamus with moderate mass effect and midline crossing **(a)**. The first image of the lower case displays a T2 weighted TSE sequence of a diffuse astrocytoma (WHO grade 2) originating from the right mesial frontal lobe **(c)**. These images are good examples of MRI-morphological similarities often found between different tumor entities, which makes further histopathological classification using conventional radiological assessment impossible. The second images of each row show the T2 SI histograms (**b**,**d**; *x*-axis: SI values in incremental order, *y*-axis: number of voxels).

Figure 1 depicts illustrative instances of cranial MRI scans obtained from individuals diagnosed with WHO grade 1 astrocytoma (upper case) and WHO grade 2 astrocytoma (lower case). Additionally, the corresponding histograms of the whole tumor T2 signal intensities (SI) are presented in the respective image series.

The findings of the descriptive analysis of T2 SI data, tumor volumes, and Ki-67 proliferation index for all examined gliomas are presented in Table 1.

**Table 1:** Tumor volume, SI histogram parameters and Ki-67 proliferation index of all investigated gliomas.

Parameters	Mean ± Standard deviation	Minimum	Maximum
Lesion volume × 10^3^ mm^3^	50.87 ± 59.00	0.72	212.51
SI_mean_	682.40 ± 186.70	374.80	1,193.00
SI_min_	177.98 ± 120.35	1	567
SI_max_	1,085.34 ± 273.52	557	1,866
SI_P10_	484.52 ± 120.53	278	854
SI_P25_	577.37 ± 155.67	312	996
SI_P75_	788.97 ± 227.61	428	1,448
SI_P90_	859.27 ± 240.62	466	1,582
Median	694.58 ± 208.54	356	1,268
Mode	725.98 ± 250.58	203	1,365
Standard deviation	145.06 ± 59.37	44.12	329.30
Kurtosis	3.03 ± 1.52	1.80	11.78
Skewness	−0.06 ± 0.64	−1.29	1.95
Entropy	5.01 ± 0.76	3.26	6.11
Ki-67%	4.55 ± 2.31	1	10

Gaussian distribution was confirmed by Shapiro–Wilk-Test for SImax, SIp75, SIp90, SImodus, SI SD, Skewness, Entropy, and Ki-67. Conversely, non-Gaussian distribution was identified for SImean, SImin, SIp10, SIp25, SImedian, Kurtosis, and tumor volume.

Consequently, an (unpaired) T-Test was employed for the comparative analysis of parameters, including SImax, SIp75, SIp90, SImodus, SI SD, Skewness, Entropy, and Ki-67, across different categories such as grade 1 and grade 2 gliomas, IDH 1 mutation-positive and negative gliomas, MGMT promoter methylated and unmethylated gliomas, as well as between oligodendrogliomas and diffuse astrocytomas. Whereas, the Mann–Whitney-U Test was utilized to assess the statistical differences in parameters SImean, SImin, SIp10, SIp25, SImedian, Kurtosis, and tumor volume among the specified groups.

Statistically significant distinctions between WHO grade 1 and grade 2 gliomas were observed for T2 SI Entropy (*p* = 0.001), tumor volume (*p* = 0.04) and Ki-67 proliferation index (*p* < 0.001). The mean values of T2 SI Entropy, tumor volume, and Ki-67 proliferation index were all markedly lower in the WHO grade I group.

Comparison of T2 SI histogram profiles of IDH 1 mutated and IDH 1 wildtype gliomas revealed significant differences for Entropy (*p* = 0.015), with higher values in case of mutated IDH 1. Whereas a subgroup analysis comparing IDH 1 mutated and IDH 1 wildtype gliomas within the WHO grade 2 cohort revealed no statistical significance (*p* = 0.281).

Moreover, noteworthy distinctions between gliomas with MGMT promoter methylation and those without were discerned for SImin (*p* = 0.019), exhibiting an elevation in unmethylated gliomas.

On the other hand, no significance could be detected comparing SI histogram parameters between oligodendrogliomas and diffuse astrocytomas.

For the sake of comprehensibility and clarity, the outcomes of the comparative statistical analyses are succinctly presented in Tables 2–5.

**Table 2:** Comparison of SI histogram parameters, Ki-67 index and lesion volume between WHO grade 1 and grade 2 gliomas.

Parameters	WHO I °	WHO II °	*p*-values
	Mean ± SD	Mean ± SD	
Lesion volume × 10^3^ mm^3^	25.58 ± 31.60	58.38 ± 63.40	**0**.**040**
SI_mean_	733.01 ± 191.89	669.19 ± 185.38	0.318
SI_min_	239.36 ± 168.78	161.90 ± 100.73	0.172
SI_max_	1,133.73 ± 254.21	1,072.67 ± 279.87	0.515
SI_P10_	503.78 ± 119.07	479.47 ± 121.60	0.492
SI_P25_	621.80 ± 148.65	565.74 ± 157.09	0.292
SI_P75_	847.73 ± 246.68	773.58 ± 222.92	0.341
SI_P90_	912.71 ± 263.27	845.27 ± 235.71	0.413
Median	762.45 ± 227.23	676.81 ± 202.50	0.229
Mode	793.01 ± 262.94	708.40 ± 247.49	0.321
Standard deviation	159.30 ± 78.08	141.33 ± 53.99	0.377
Kurtosis	3.80 ± 2.76	2.83 ± 0.93	0.227
Skewness	−0.09 ± 0.05	−0.04 ± 0.58	0.863
Entropy	4.37 ± 0.88	5.18 ± 0.64	**0**.**001**
Ki-67%	2.5 ± 1.65	5.11 ± 2.16	**<0**.**001**

Statistically significant *p*-values are given in bold.

**Table 3:** Comparison of SI histogram parameters between IDH 1 mutated and IDH 1 wildtype gliomas.

Parameters	IDH 1 mutation	IDH wildtype	*p*-values
	Mean ± SD	Mean ± SD	
SI_mean_	675.0 ± 165.0	711.7 ± 227.3	0.530
SI_min_	159.8 ± 105.2	213.7 ± 150.8	0.159
SI_max_	1,079.0 ± 242.9	1,109 ± 348.0	0.732
SI_P10_	488.5 ± 116.7	501.5 ± 140.2	0.730
SI_P25_	573.2 ± 144.9	597.2 ± 179.75	0.624
SI_P75_	777.6 ± 195.8	827.1 ± 281.2	0.485
SI_P90_	844.0 ± 199.5	914.59 ± 309.5	0.347
Median	684.5 ± 180.3	719.5 ± 250.0	0.584
Mode	717.9 ± 228.5	750.5 ± 296.1	0.679
Standard deviation	138.9 ± 43.5	156.0 ± 78.5	0.336
Kurtosis	2.88 ± 01.00	2.87 ± 0.79	0.965
Skewness	−0.09 ± 0.62	−0.001 ± 0.46	0.366
Entropy	5.22 ± 0.62	4.65 ± 0.92	**0**.**015**
Entropy (WHO II °)	5.23 ± 0.63	4.95 ± 0.74	0.281

Statistically significant *p*-values are given in bold.

**Table 4:** Comparison of SI histogram parameters between MGMT methylated and MGMT unmethylated gliomas.

Parameters	MGMT methylated	MGMT unmethylated	*p*-values
	Mean ± SD	Mean ± SD	
SI_mean_	699.5 ± 215.5	746.3 ± 176.2	0.536
SI_min_	144.8 ± 114.1	258.0 ± 146.6	**0**.**019**
SI_max_	1,108.1 ± 319.1	1,181.64 ± 269.0	0.514
SI_P10_	498.4 ± 138.0	539.4 ± 133.4	0.419
SI_P25_	592.0 ± 181.0	624.7 ± 151.4	0.608
SI_P75_	811.0 ± 259.6	857.6 ± 211.6	0.608
SI_P90_	881.7 ± 271.3	945.3 ± 221.1	0.504
Median	710.6 ± 238.1	751.7 ± 194.6	0.623
Mode	750.2 ± 282.4	772.27 ± 270.3	0.830
Standard deviation	146.2 ± 59.7	161.5 ± 55.2	0.479
Kurtosis	2.74 ± 00.56	3.09 ± 1.52	0.971
Skewness	−0.12 ± 0.49	0.18 ± 0.73	0.201
Entropy	5.21 ± 0.73	4.82 ± 0.71	0.151

Statistically significant *p*-values are given in bold.

**Table 5:** Comparison of SI histogram parameters between WHO 2 oligodendrogliomas and diffuse astrocytomas.

Parameters	Oligodendrogliomas	Diffuse Astrocytomas	*p*-values
	Mean ± SD	Mean ± SD	
SI_mean_	612.77 ± 112.84	689.21 ± 202.83	0.245
SI_min_	183.09 ± 85.28	154.39 ± 10,592.8	0.493
SI_max_	1,030 ± 212.81	1,087.81 ± 301.74	0.616
SI_P10_	451 ± 83.16	489.57 ± 132.30	0.372
SI_P25_	521 ± 100.97	581.61 ± 171.26	0.276
SI_P75_	703.55 ± 130.33	798.42 ± 244.57	0.230
SI_P90_	769.55 ± 129.92	872.14 ± 259.72	0.219
Median	616 ± 126.34	698.39 ± 221.10	0.251
Mode	658.73 ± 151.69	726.03 ± 273.52	0.445
Standard deviation	122.05 ± 25.27	148.18 ± 59.88	0.171
Kurtosis	2.73 ± 0.58	2.87 ± 1.03	0.888
Skewness	−0.01 ± 0.41	−0.06 ± 0.63	0.822
Entropy	5.12 ± 0.55	5.20 ± 0.68	0.733

**Figure 2:**
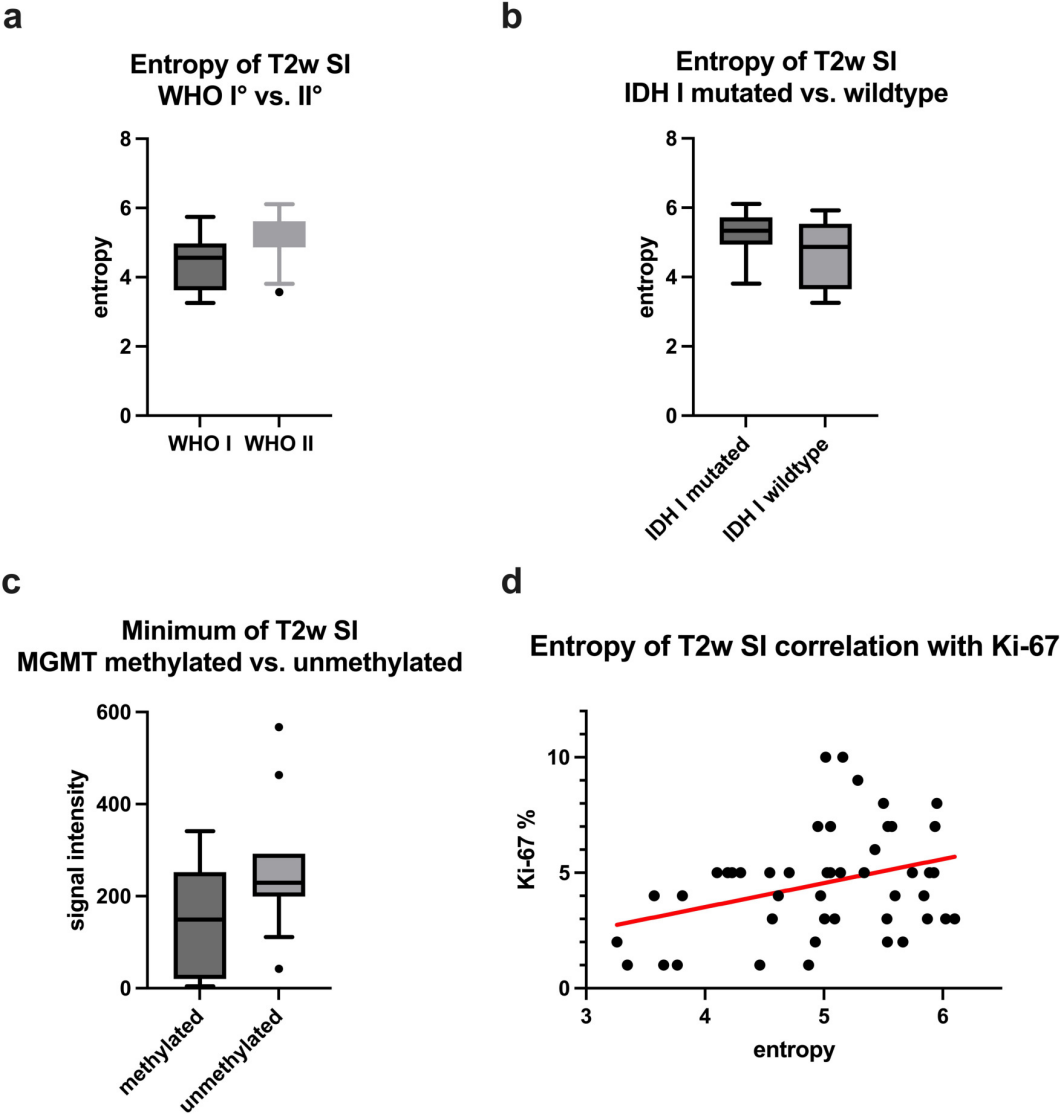
Figure 2 provides boxplots of statistically significant differences between the T2 SI entropy of grade 1 and grade 2 gliomas **(a)** and of IDH 1 mutated and IDH 1 wildtype gliomas **(b)**. A boxplot demonstrating the significant difference of T2 SI minimum between MGMT promoter methylated and unmethylated gliomas is displayed in **(c)**. The last image **(d)** shows a moderate correlation between T2 SI entropy and the proliferation index Ki-67.

Figures 2a–c provides box plots illustrating significant SI histogram profile parameters related to WHO grade, IDH I mutation, and MGMT methylation status.

Potential associations among SImax, SIp75, SIp90, SImodus, SI SD, Skewness, Entropy, and Ki-67 were explored through the calculation of Pearson's Correlation Coefficient. Spearman-Rho Rank-Order Correlation was employed to investigate potential associations between SImean, SImin, SIp10, SIp25, SImedian, Kurtosis, and Ki-67. A significant, albeit moderate, correlation was observed between Ki-67 and Entropy (*r* = 0.341, *p* = 0.019). The comprehensive results of the correlative analyses are summarized in Table 6.

**Table 6:** Correlations between SI histogram parameters and Ki-67 in all investigated gliomas.

Parameters	Ki-67
SI_mean_	*r* = −0.179
	*p* = 0.216
SI_min_	*r* = −0.147
	*p* = 0.315
SI_max_	*r* = −0.004
	*p* = 0.980
SI_P10_	*r* = −0.135
	*p* = 0.355
SI_P25_	*r* = −0.165
	*p* = 0.258
SI_P75_	*r* = −0.195
	*p* = 0.180
SI_P90_	*r* = −0.196
	*p* = 0.177
Median	*r* = −0.177
	*p* = 0.221
Mode	*r* = −0.156
	*p* = 0.286
Standard deviation	*r* = −0.181
	*p* = 0.214
Kurtosis	*r* = 0.107
	*p* = 0.465
Skewness	*r* = 0.101
	*p* = 0.490
Entropy	*r* = 0.341
	***p*** **=** **0.019**

Statistically significant *p*-values are given in bold.

Additionally, the scatter plot graphically depicting the association between T2 SI Entropy and Ki-67 is presented in Figure 2d.

**Figure 3:**
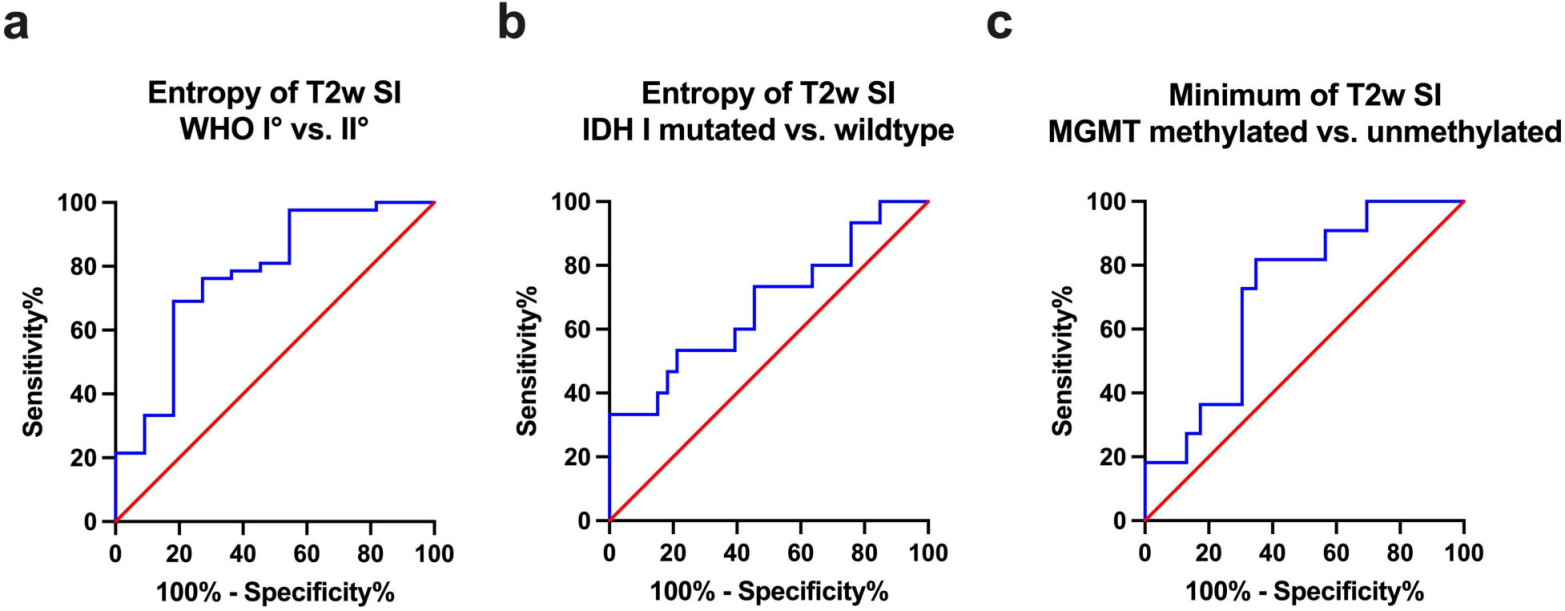
Figure 3 provides the receiver operating characteristics (ROC) curve of T2 SI entropy in terms of differentiating grade 1 and grade 2 gliomas **(a)** as well as in terms of differentiating IDH 1 mutated and IDH 1 wildtype gliomas **(b)**. Image **(c)** shows the ROC curve of T2 SImin concerning the differentiation of MGMT promoter methylated and unmethylated gliomas.

Moreover, Area Under the Curve (AUC) values were computed for each of the assessed parameters that demonstrated statistically significant differences. The obtained values, along with their confidence intervals (CI), are as follows: SI Entropy WHO 1 vs. 2 (AUC = 0.775, [CI: 0.608–0.942], *p* = 0.005), SI Entropy IDH 1 mutated vs. wildtype (AUC = 0.677, [CI: 0.504–0.850], *p* = 0.052), SImin MGMT methylated vs. unmethylated (AUC = 0.715, [CI: 0.539–0.892], *p* = 0.045). The corresponding Receiver Operating Characteristic (ROC) curves are presented in Figure 3.

Finally, Youden's Index for SI Entropy was calculated to determine the most promising cutoff value for distinguishing WHO grade 1 and 2 gliomas, revealing an Entropy value of 4.99 and above to indicate WHO grade 2 with a sensitivity of 69.05% and a specificity of 81.82%.

## 4 Discussion

In this retrospective investigation we systematically analyzed T2 weighted MRI images, using a quantitative approach by histogram profiling, to elucidate potential applications in the *in-vivo* classification of tumor grades and the prediction of prognostically relevant molecular parameters. Not surprisingly, we detected significant differences in the comparative statistical analysis between WHO grade 1 and grade 2 gliomas concerning Ki-67 proliferation index and tumor volume with higher volume and higher proliferative activity in the grade 2 group, as estimating the proliferation is part of the pathological grading system and a higher proliferation rate inevitably leads to faster tumor growth.

As introduced above, a histogram is a graphical representation of the frequency distribution of a dataset. It provides a visual summary of the data, making it easier to identify specific patterns or characteristics. Different parameters are routinely used in the context of HA to describe the data characteristics. There are those, which reflect the center and location of the histogram in the data continuum like median, mean, mode, minimum, maximum and the percentiles, sometimes referred as first order parameters and those indicating the shape and spread of the histogram like skewness, kurtosis and entropy, sometimes called second order parameters. First order histogram characteristics have the same unit as the investigated variable, in our case T2 signal intensity, and thus depend directly on the measured values. For example, higher T2 signal of the respective tumor inevitably leads to higher first order histogram parameters in general, regardless of the morphological tumor structure and tumor heterogeneity. Second order histogram characteristics, on the other hand, are unit independent. They represent different aspects of the data distribution independently of the absolute values and should therefore be more suitable for the assessment of tumor heterogeneity using conventional T2 weighted MRI images. This postulation is in line with the present study results of the comparative statistics. None of the first order characteristics achieved significance between grade 1 and grade 2 gliomas, whereas T2 SI Entropy as second order histogram parameter was significantly lower in the grade 1 group, with strong significance (*p* = 0.001). Similar results have been presented in previous studies, showing histogram Entropy to be increased in case of higher tumor grades and malignant neoplasia (13, 14, 26–30). In the context of histogram analysis, entropy is a measure of the randomness or disorder in a distribution of data values. It can be used to quantify the amount of information or uncertainty in a histogram. The entropy (H) of a discrete probability distribution is typically computed using the formula:

$$H(x)=\;-\;\sum_{i=1}^{n}\;P(x_{i})\times \;\mathrm{lo}g_{2}(P(x_{i})),$$

where *n* is the number of intensity levels in the histogram and $P(x_{i})$ is the probability of occurrence of the *i*-th intensity level. Entropy is high when the distribution is more uniform or when there is no dominant intensity level, indicating greater uncertainty. On the other hand, low entropy suggests a more peaked or concentrated distribution with less uncertainty. Applied to our case of T2 weighted MRI images, this would mean that the entropy would be minimal if the measured lesion had only a single signal intensity (for example an idealized homogenous cyst). In contrast, the more morphologically complex and diverse the lesion, consisting of many different signal intensities without a concentration of individual intensities, the higher the corresponding T2 SI entropy would be. Understanding these underlying mathematical principles and transferring them to HA of MRI data, one must conclude that particularly histogram entropy should be a very suitable parameter for assessing the heterogeneity of a lesion. This conclusion is also supported by the study results of the statistical correlation analysis, in which the T2 SI entropy correlates positively with the proliferation index Ki-67 (r = 0.341, *p* = 0.019). Since the tumor grade is by definition directly linked to the mitotic activity of the tumor tissue and higher proliferation in turn leads to increased tissue heterogeneity, we consider the correlation between entropy and Ki-67 to be further confirmation of the assumption that entropy could be a possible heterogeneity marker. A number of recent studies have also demonstrated a statistical correlation between the histogram entropy of various MRI data and the proliferation index Ki-67, thus supporting this hypothesis (13, 15, 20, 31–33).

In this study we also investigated whether HA of T2 SI enables the prediction of the IDH 1 mutation and the MGMT promoter methylation status of the respective glioma. Protein-truncating mutation in isocitrate dehydrogenase 1 (IDH 1) or in isocitrate dehydrogenase 2 (IDH 2) is associated with a more favorable outcome and a longer overall survival in case of low-grade astrocytomas and oligodendroglial tumors (34–36). In addition, methylation of the promoter of O-6-methylguanine-DNA methyltransferase (MGMT) gene limits the ability of the tumor cells to repair DNA damage caused by chemotherapy and radiation, resulting in increased survival time (37–39). In the comparative statistics we detected significant higher T2 SI entropy values in the IDH 1 mutant group compared to the wildtype group, even though the ROC analysis was not quite significant. Since all grade 1 gliomas in our study cohort were pilocytic astrocytomas and they usually do not carry mutations in the IDH 1 gene (in our investigation only one out of eleven) and the major part of the grade 2 glioma cohort were IDH 1 mutants (42 IDH 1 mutants vs. 8 IDH 1 wildtypes) and thus comparing IDH 1 wildtype with IDH 1 mutant in our study effectively means comparing grade 1 with grade 2 glioma, we believe this result not to be an independent effect of the IDH 1 mutation status but rather an overlapping effect of the tumor grade. The subgroup analysis, comparing IDH 1 mutant and IDH 1 wildtype glioma within the WHO grade 2 cohort, supported this suspicion, although the probability of a type II error is likely to be relatively high due to the small number of grade 2 IDH wildtype tumors (8 in total). Furthermore, MGMT promoter methylated gliomas showed significantly lower minimal T2 signal intensities (SImin) in contrast to unmethylated entities. Interestingly, a similar result was obtained in a previous histogram study for the MGMT status of LGG concerning minimum values of apparent diffusion coefficient (ADC) (14). The MGMT promoter methylated LGG in this study had significant lower ADCmin values compared to the unmethylated gliomas and it was also the only histogram parameter that reached significance in the comparative statistics. Nevertheless, as there are no other histogram studies of simple MRI signal intensities in gliomas, especially not with investigation of the MGMT promoter methylation status, it is not yet possible to assess whether the SImin values represent a true MGMT promoter methylation status-dependent phenotype. Furthermore, it must also be taken into account that the MRI images were not normalized, which may have influenced the results of the first-order HA parameters. Further studies with bigger samples and normalized SI datasets are needed in this context to clarify the significance of this finding.

Additionally, we compared T2 SI histogram parameters of grade 2 glioma with and without codeletion of chromosomal arms 1p and 19q. None of the HA parameters achieved statistical significance in this context. But this result could be due to the relatively low number of oligodendrogliomas in this study (12 in total) with consequently high probability for false negative results. Whether this result is a reflection of the possibly comparable tumor heterogeneity on the micro- and macroscopic scale cannot be confirmed with certainty based on the results of this study.

Our study exhibits certain limitations that warrant consideration. Primarily, it is characterized as a retrospective investigation with a relatively small number of patients, especially in the subgroups, and thus potentially statistically underpowered. Therefore, the non-significant results can only be interpreted to a limited extent. It should also be mentioned that, due to the exploratory nature of this study, we combined circumscribed and diffuse gliomas in our cohort and thus used the term “low-grade glioma” in a broader sense compared to the latest edition of the WHO classification of tumors of the CNS from 2021. Additionally, the exclusive availability of data derived from 1.5-Tesla MRI systems inherently results in diminished signal-to-noise ratios within the MRI data. This necessitates the acquisition of MRI images with a reduced pixel matrix, consequently leading to a diminution in spatial information when compared to examinations with a higher field strength. Also, the acquisition of T2 images with a slice thickness of 5 mm could alter the histogram caused by partial volume effects at the tumor margins. Moreover, due to partially missing data of the patient sample, especially concerning the MGMT-methylation status, statistical bias and therefore lower statistical power and accuracy may have occurred in these instances. Furthermore, related to the preliminary nature of our investigation, the familywise alpha inflation due to serial hypothesis tests in the statistical analysis was not controlled.

Finally, it was only a single center study using MRI devices from the same manufacturer. Although the vast majority of patients were examined using the same MRI scanner, further studies are required to clarify whether the type of MRI device and manufacturer as well as the examination parameters have an influence on the histogram parameters and thus to validate the robustness and reliability of the HA of simple morphological MRI data as method to assess tumor heterogeneity.

## 5 Conclusion

Histogram profiling of T2 weighted morphological MRI data encompasses the derivation of first and second order characteristics through distinct parameters. Our investigation demonstrates the potential of T2 SI entropy as second order parameter in distinguishing grade 1 from grade 2 gliomas and in reflecting the proliferative activity denoted by Ki-67 expression. We postulate that entropy, in particular, is a promising HA parameter in terms of assessing tumor heterogeneity and should be subject of further imaging studies. For example, it is quite conceivable that entropy could also be a suitable parameter as part of an AI-supported approach with appropriately trained deep neural networks.

## Data Availability

The original contributions presented in the study are included in the article/Supplementary Material, further inquiries can be directed to the corresponding author.
